# Supplementary material for: Acute Respiratory Tract Infection and 25-Hydroxyvitamin D Concentration: A Systematic Review and Meta-Analysis
Source: Int J Environ Res Public Health. 2019 Aug 21;16(17):3020. doi: 10.3390/ijerph16173020 (PMC6747229; doi:10.3390/ijerph16173020)
Supplement: Supplementary file 1 [file ijerph-16-03020-s001.zip › Supplementary files/File S1.docx]

**Supplementary file S1. Search strategies.**

**PUBMED**: “25OHD”[tiab] OR “25(OH)D” [tiab] OR “hypovitaminosis D” [tiab]) AND ("respiratory tract infections"[mesh] OR "respiratory tract infection"[tiab] OR "respiratory infection"[tiab] OR “respiratory disease” [tiab] OR "respiratory tract infections"[tiab] OR "respiratory infections"[tiab] OR “respiratory diseases” [tiab] OR "respiratory tract infectio*"[tiab] OR "respiratory infectio*"[tiab] OR “respiratory diseas*” [tiab] OR "pneumonia"[tiab] OR "influenza"[tiab] OR "bronchiolitis"[tiab] OR "common cold"[tiab])

**EMBASE:** 'vitamin D'/exp OR 'vitamin D':ab,ti OR '25 hydroxyvitamin D'/exp OR '25 hydroxyvitamin D':ab,ti OR 'vitamin D deficiency'/exp AND 'respiratory tract infection'/exp OR 'respiratory tract infection':ab,ti OR 'respiratory tract disease'/exp OR 'pneumonia':ab,ti OR 'bronchiolitis':ab,ti OR 'influenza':ab,ti OR 'common cold':ab,ti AND ('case control study'/de OR 'cohort analysis'/de OR 'observational study'/de OR 'prospective study'/de) AND ([adolescent]/lim OR [adult]/lim) AND [english]/lim.
