# Supplementary material for: Acute Respiratory Tract Infection and 25-Hydroxyvitamin D Concentration: A Systematic Review and Meta-Analysis
Source: Int J Environ Res Public Health. 2019 Aug 21;16(17):3020. doi: 10.3390/ijerph16173020 (PMC6747229; doi:10.3390/ijerph16173020)
Supplement: Supplementary file 1 [file ijerph-16-03020-s001.zip › Supplementary files/Table S1.docx]

Table S1. Quality assessment based on the Newcastle-Ottawa Scale (NOS).

| **First Author (Published Year)** | **Total Score** | **Selection** | **Comparability** | **Outcome** |
| --- | --- | --- | --- | --- |
| ***Case control studies (n = 3)*** | | Max score  possible = 5 | Max score  possible = 2 | Max score  possible = 3 |
| Javanovich, A.J. (2014) | 7 | *** (baab) | * (b) | *** (baa) |
| Nanri, A. (2017) | 7 | **** (baaa) | * (ab) | ** (bab) |
| Mamani, M. (2017) | 8 | ***** (aaaa) | * (a) | ** (aac) |
| ***Cross-sectional studies (n = 13)*** | | Max score  possible = 4 | Max score  possible = 2 | Max score  possible = 3 |
| Ginde, A.A. (2009) | 7 | *** (bbca) | ** (ab) | ** (ca) |
| Berry, D.J. (2011) | 8 | **** (bbaa) | ** (ab) | ** (ca) |
| Quraishi, S.A. (2013) | 7 | *** (abca) | ** (ab) | ** (ca) |
| Robertsen, S. (2014) | 7 | **** (bbaa) | * (a) | ** (ca) |
| Pletz, M.W. (2014) | 9 | **** (abaa) | ** (ab) | *** (ba) |
| Monlezun, D. (2015) | 7 | *** (bbca) | **(ab) | ** (ca) |
| Kim, H.J. (2015) | 7 | *** (bbca) | * (a) | *** (ba) |
| Khalid, A.N. (2015) | 8 | **** (baca) | ** (ab) | ** (ca) |
| Lu, D. (2017) | 6 | ** (cbca) | * (b) | *** (ba) |
| Scullion, L. (2018) | 7 | **** (baaa) | * (a) | ** (ca) |
| Brance, M. (2018) | 6 | *** (bbca) | 0 | *** (aa) |
| Rafiq, R. (2018) | 7 | *** (bbca) | **(ab) | *** (ba) |
| Yaghoobi, M.H. (2019) | 6 | ** (bbac) | * (b) | *** (aa) |
| ***Prospective cohort studies (n = 8)*** | | Max score  possible = 4 | Max core  possible = 2 | Max score  possible = 4 |
| Laaksi, I. (2007) | 8 | *** (baab) | * (a) | **** (bab) |
| Sabetta, J.R. (2010) | 6 | ** (caab) | 0 | **** (aab) |
| Leow, L. (2011) | 8 | **** (aaaa) | 0 | **** (baa) |
| Remmelt, H. (2012) | 10 | **** (baaa) | ** (ab) | **** (baa) |
| Porojnicu, A.C. (2012) | 6 | ** (cabb) | 0 | **** (bab) |
| Aregbesola, A. (2013) | 9 | *** (baab) | ** (ab) | **** (bab) |
| He, C.S. (2013) | 7 | **** (aaaa) | 0 | *** (cab) |
| Holter, J.C. (2016) | 9 | **** (baaa) | * (ab) | **** (bab) |
| The maximum score for the Newcastle-Ottawa Scale is 10 (9 for cross-sectional studies). The number of * indicates the total score for each section, the a,b and c correspond to the answer for each question. | | | | |
