# Supplementary material for: Acute Respiratory Tract Infection and 25-Hydroxyvitamin D Concentration: A Systematic Review and Meta-Analysis
Source: Int J Environ Res Public Health. 2019 Aug 21;16(17):3020. doi: 10.3390/ijerph16173020 (PMC6747229; doi:10.3390/ijerph16173020)
Supplement: Supplementary file 1 [file ijerph-16-03020-s001.zip › Supplementary files/Table S2.docx]

Table S2. Studies exlcuded from the meta-analysis and main findings.

| **First Author (Published Year)** | **Reasons for Exclusion from Meta-Analysis** | **Main Findings** |
| --- | --- | --- |
| *Study reporting the association between 25(OH)D concentration and risk of ARTI* | | |
| Porojnicu, A.C. (2012) | Reported correlation | There was no significant correlation between serum concentration of 25(OH)D and any infection; Spearman correlation coefficient −0.12, *p* = 0.2 |
| Scullion, L. (2018) | Reported the number (%) of people with ARTI, comparing the rugby players versus the rowers in summer and winter | More people reported ARTI in winter than in summer (52% vs. 36%, *p* = 0.322); there was no significant difference in the mean (SD) 25(OH)D concentration between people with and without ARTI (in summer: 111.0 (20.3) nmol/L vs. 111.3 (24.7) nmol/L, *p* = 0.965; nor in winter (85.2 (16.7) nmol/L vs. 93.4 (19.9) nmol/L, *p* = 0.123) |
| *Study reporting the association between 25(OH)D concentration and ARTI severity* | | |
| Scullion, L. (2018) | Reported mean (SD) duration of ARTI, comparing summer and winter | People who had ARTI in winter had more prolonged illness than those who reported ARTI in summer (mean (SD) was 6.9(4.3) vs. 4.8(3) days) |
| Yaghoobi, M.H. (2019) | Reported mean (SD) 25(OH)D concentration, comparing people with severe vs non-severe URTI; and mean (SD) severity score, comparing 25(OH)D category of <75 nmol/L versus ≥75 nmol/L | 25(OH)D concentration was not associated with severity of ventilator-associated pneumonia |
| Brance, M. (2018) | Reported mean (SD) 25(OH)D concentration, comparing people low versus intermediate/high risk of CURB-65 | There was no difference in the mean (SD) 25(OH)D concentration according to CURB-65 (low risk 0–1: 29.8 (18.8) nmol/L; intermediate/high risk 2–5: 29.0 (18.3) nmol/L |
| Lu, D. (2017) | Reported mean (SD) length of hospital stay, comparing 25(OH)D category of <25 nmol/L versus ≥25 nmol/L | Significant inverse correlation between 25(OH)D concentration and length of hospital stay (*r* = −0.413, *p* = 0.003), mean (SD) was 26.2 (15.6) vs. 15.5 (11.1) (*p* = 0.012) |
| Pletz, M.W. (2014) | Reported mean (SD) 25(OH)D concentration, comparing people with versus without hospitalisation | 25(OH)D concentration was lower in people who needed hospitalisation compared to people who were not hospitalised, mean (SD) was 32.0 (19.5) vs. 40.5 (25.0) nmol/L |
| Robertsen, S. (2014) | Reported trend for increasing duration of illness according to 25(OH)D concentration | There was no trend for increasing or decreasing duration of illness across quartiles of 25(OH)D concentration |
| He, C-S. (2013) | Reported median (Q1–Q3) symptom severity score and duration of the illness, comparing 4 categories of 25(OH)D concentration | The median symptom-severity score in the deficient group (25(OH)D concentration < 30 nmol/L) was significantly higher than the other groups (*p* = 0.013), and borderline singificant difference was found in the median duration of episodes (*p* = 0.059) |
| Sabetta, J.R. (2010) | Reported median (range) duration (days) of ARTI, comparing 25(OH)D concentration < 95 nmol/L versus ≥ 95 nmol/L | No significant difference in the duration of ARTI between 25(OH)D concentration ≥ 95 nmol/L and 25(OH)D concentration < 95 nmol/L, medians (ranges) were 6 (2–27) vs. 6 (2–8) |
| Laaksi, I. (2007) | Reported incidence rate ratio | Significant inverse correlation between 25(OH)D concentration and number of days absence from duty, incidence rate ratio was 1.63 (1.15–2.24); comparing 25(OH)D concentration <40 nmol/L vs. ≥40 nmol/L |
| Abbreviations: ARTI = acute respiratory tract infection; SD = standard deviation; Q1–Q3: Quartile1–Quartile3. | | |
